# Dr. Roger W. Miller

**Published:** 1913-03

**Authors:** 


Dr. Roger W. Miller, Bellevue, 1870, died suddenly at his home in Castile, N. Y., February 22.

